# Supplementary material for: HIVprotI: an integrated web based platform for prediction and design of HIV proteins inhibitors
Source: J Cheminform. 2018 Mar 9;10:12. doi: 10.1186/s13321-018-0266-y (PMC5845081; doi:10.1186/s13321-018-0266-y)
Supplement: Supplementary file 2 — Additional file 2. Source code of HIVProtI web server. [file 13321_2018_266_MOESM2_ESM.zip › HIVprotI_Source-code/hivprotI/weka-3-6-6/doc/weka/classifiers/trees/j48/EntropyBasedSplitCrit.html]

EntropyBasedSplitCrit


---


|  |  |  |
| --- | --- | --- |
| **Overview** \| **Package** \| **Class** \| **Tree** \| **Deprecated** \| **Index** \| **Help** \| **Weka's home** | |  |
| **PREV CLASS**   **NEXT CLASS** | **FRAMES**    **NO FRAMES**     **All Classes** |  |
| SUMMARY: NESTED \| FIELD \| CONSTR \| METHOD | DETAIL: FIELD \| CONSTR \| METHOD |  |


---


## weka.classifiers.trees.j48 Class EntropyBasedSplitCrit

```
java.lang.Object
  weka.classifiers.trees.j48.SplitCriterion
      weka.classifiers.trees.j48.EntropyBasedSplitCrit
```

**All Implemented Interfaces:**: java.io.Serializable, RevisionHandler

**Direct Known Subclasses:**: EntropySplitCrit, GainRatioSplitCrit, InfoGainSplitCrit

---

``` public abstract class EntropyBasedSplitCrit extends SplitCriterion ```

"Abstract" class for computing splitting criteria
based on the entropy of a class distribution.

**Version:**
:   $Revision: 1.8 $

**Author:**
:   Eibe Frank (eibe@cs.waikato.ac.nz)

**See Also:**: Serialized Form

---

**Constructor Summary**

`EntropyBasedSplitCrit()`


| **Method Summary** | |
| --- | --- |
| `double` | `logFunc(double num)`             Help method for computing entropy. |
| `double` | `newEnt(Distribution bags)`             Computes entropy of distribution after splitting. |
| `double` | `oldEnt(Distribution bags)`             Computes entropy of distribution before splitting. |
| `double` | `splitEnt(Distribution bags)`             Computes entropy after splitting without considering the class values. |

**Methods inherited from class weka.classifiers.trees.j48.SplitCriterion**

`splitCritValue, splitCritValue, splitCritValue, splitCritValue`

**Methods inherited from class java.lang.Object**

`equals, getClass, hashCode, notify, notifyAll, toString, wait, wait, wait`

**Methods inherited from interface weka.core.RevisionHandler**

`getRevision`

**Constructor Detail**

### EntropyBasedSplitCrit

```
public EntropyBasedSplitCrit()
```


**Method Detail**

### logFunc

```
public final double logFunc(double num)
```

:   Help method for computing entropy.

---


### oldEnt

```
public final double oldEnt(Distribution bags)
```

:   Computes entropy of distribution before splitting.

---


### newEnt

```
public final double newEnt(Distribution bags)
```

:   Computes entropy of distribution after splitting.

---


### splitEnt

```
public final double splitEnt(Distribution bags)
```

:   Computes entropy after splitting without considering the
    class values.


---


|  |  |  |
| --- | --- | --- |
| **Overview** \| **Package** \| **Class** \| **Tree** \| **Deprecated** \| **Index** \| **Help** \| **Weka's home** | |  |
| **PREV CLASS**   **NEXT CLASS** | **FRAMES**    **NO FRAMES**     **All Classes** |  |
| SUMMARY: NESTED \| FIELD \| CONSTR \| METHOD | DETAIL: FIELD \| CONSTR \| METHOD |  |


---
